# Association between a Novel Mutation in *SLC20A2* and Familial Idiopathic Basal Ganglia Calcification

**DOI:** 10.1371/journal.pone.0057060

**Published:** 2013-02-20

**Authors:** Yang Zhang, Xianan Guo, Anhua Wu

**Affiliations:** 1 Research Center for Medical Genomics, Key Laboratory of Medical Cell Biology, Ministry of Education, College of Basic Medical Science, China Medical University, Shenyang, China; 2 Department of Neurosurgery, the First Affiliated Hospital of China Medical University, Shenyang, China; University of Bonn, Institut of Experimental Hematology and Transfusion Medicine, Germany

## Abstract

Familial idiopathic basal ganglia calcification (FIBGC) is a rare, autosomal dominant disorder involving bilateral calcification of the basal ganglia. To identify gene mutations related to a Chinese FIBGC lineage, we evaluated available individuals in the family using CT scans. DNA was extracted from the peripheral blood of available family members, and both exonic and flanking intronic sequences of the *SLC20A2* gene were amplified by PCR and then sequenced. Non-denaturing polyacrylamide gel electrophoresis (PAGE) was used to confirm the presence of mutations. Allele imbalances of the *SLC20A2* gene or relative quantity of *SLC20A2* transcripts were evaluated using qRT-PCR. A novel heterozygous single base-pair deletion (c.510delA) within the *SLC20A2* gene was identified. This deletion mutation was found to co-segregate with basal ganglia calcification in all of the affected family members but was not detected in unaffected individuals or in 167 unrelated Han Chinese controls. The mutation will cause a frameshift, producing a truncated SLC20A2 protein with a premature termination codon, most likely leading to the complete loss of function of the SLC20A2 protein. This mutation may also lead to a reduction in *SLC20A2* mRNA expression by approximately 30% in cells from affected individuals. In conclusion, we identified a novel mutation in *SLC20A2* that is linked to FIBGC. In addition to the loss of function at the protein level, decreasing the expression of *SLC20A2* mRNA may be another mechanism that can regulate *SLC20A2* function in IBGC individuals. We propose that the regional expression pattern of SLC20A1 and SLC20A2 might explain the unique calcification pattern observed in FIBGC patients.

## Introduction

Calcification of basal ganglia is not uncommon. In clinical settings, this situation can be found secondary to various pathologic processes including hypoparathyroidism, infections, brain tumors, and radiation damage [1]. However, the calcification of basal ganglia may also occur as an idiopathic condition. This is known as familial idiopathic basal ganglia calcification (FIBGC) or Fahr disease [2]. Although rare, FIBGC has attracted the interest of neuroscientists ever since Fahr first described it in 1930 [3]. This unique clinical entity has often been associated with neurobehavioral and cognitive changes [1], [2]. The identification of its genetic background may provide an explanation for the formation of abnormal intracerebral calcifications and provide insight into the different pathways that lead to dysfunction of the basal ganglia.

FIBGC is often presented as a disorder with autosomal dominant inheritance. Its pathogenetic basis has been linked to chromosomes such as 14q13, 2q37, and 8q21.1-q11.23 [4]–[6]. Recently, Wang et al. sequenced the 8q21.1-q11.23 locus and confirmed that loss of function of SLC20A2, which is a type III sodium-dependent phosphate transporter, is the molecular basis for a large subset of IBGC [7]. They suggested that disturbed regional Pi homeostasis is the primary pathophysiologic mechanism of IBGC. However, familial IBGC has been reported to be genetically heterogeneous [8]. Further investigation of other genetic mutations may be necessary for fully understanding its molecular background.

## Methods

### Study subjects and MRS evaluation

**Figure 1:**
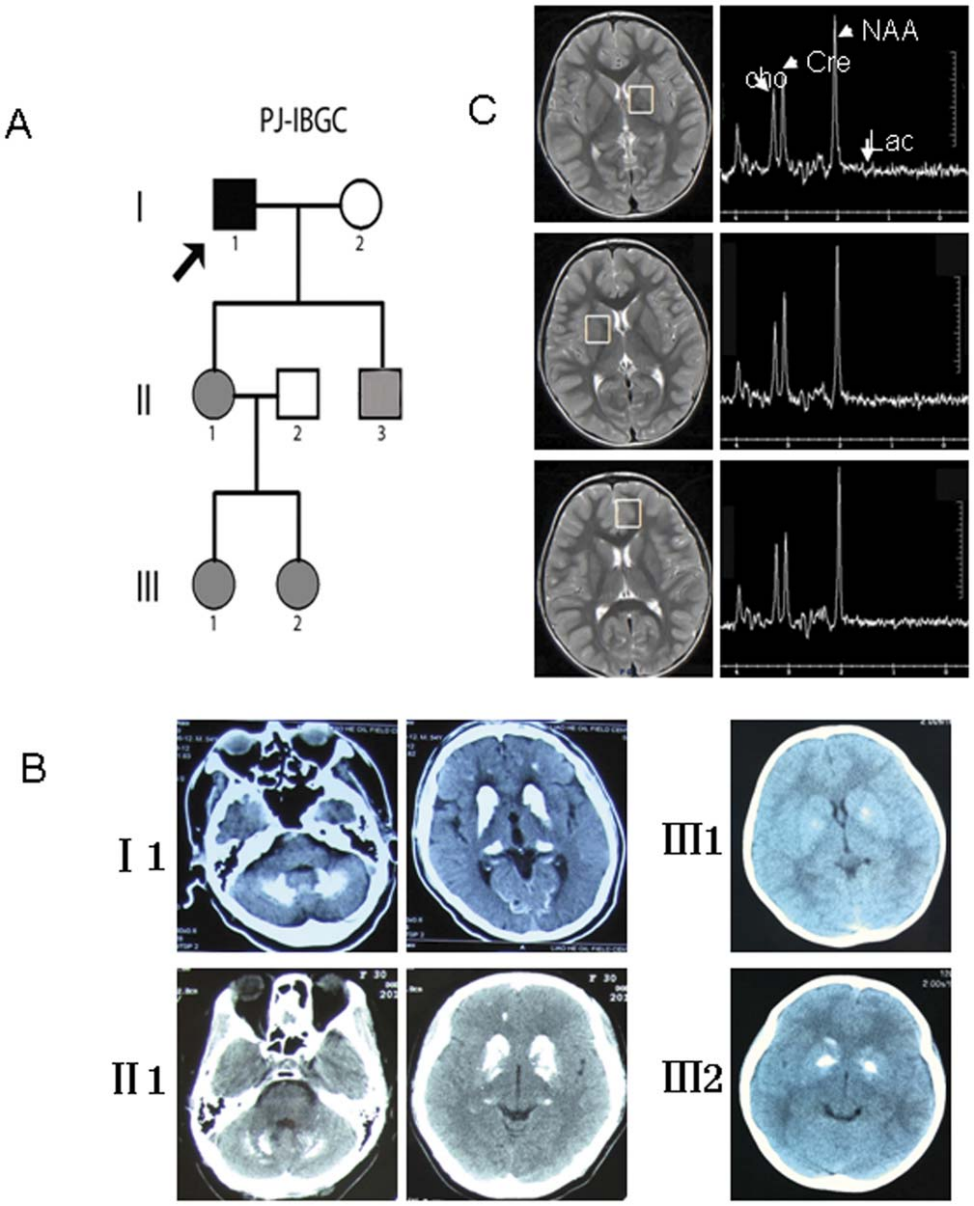
Study subjects and imaging evaluation. (A) Pedigree of family PJ-IBGC. The proband is indicated by an arrow. Filled symbols represent affected individuals, including both symptomatic (black) and asymptomatic (gray). (B) CT scan of the affected individuals. (C) MRS examination of a 4-year-old girl. I 1: a 54-year-old man; II 1: a 37-year-old woman; II:3: a 24-year-old man; III 1: an 11-year-old girl; III 2: a 4-year-old girl.

A Chinese family affected by FIBGC with an autosomal dominant pattern of inheritance is depicted in Fig. 1A. The 4-year-old girl received a CT scan because her mother had found her convulsing after crying. At the mother's request, we performed the CT scans on her other daughter as well. There were 5 affected individuals in the 3 generations (Fig. 1A). In this study, the term “affected individuals” refers to both symptomatic and asymptomatic family members with calcification on brain CT scans, except II:3, whose CT image was not available, but genetic analysis confirmed the existence of FIBGC related *SLC20A2* mutation, as in other affected members of this family (Fig. 1B). Written informed consent was obtained according to the Declaration of Helsinki, and the ethical committee of China Medical University approved this study.

The 4-year-old girl, who had minimal calcification of the basal ganglia area, also received a magnetic resonance spectroscopy (MRS) evaluation. Images were obtained using an 8-channel brain-phased array coil and a 3.0-T imaging system (Signa; GE Medical Systems, Milwaukee, WI, U.S.A.). Single-voxel spectroscopy was performed with PRESS technique (echo time 144 ms, repetition time 1500 ms); 2 cm-sided voxels were located in the basal ganglia and left frontal cortex. The basal ganglia voxels centered on the internal part of the globus pallidus, which showed minimal calcification. Peaks of cerebral metabolites were identified according to their chemical shifts. With a TE of 144 ms, lactate was defined as inverted peaks (doublet at 1.28 and 1.38 ppm), and lipid levels were found to be suppressed.

### Analysis of *SLC20A2* mutation

Peripheral blood samples were collected from the available members of the Chinese family with IBGC after informed consent was provided by each. Genomic DNA was extracted using a universal genomic DNA extraction kit (TaKaRa, Japan). All 11 exons and the flanking intronic sequences of the *SLC20A2* gene (Genbank accession number: NG_032161) were amplified using polymerase chain reaction (PCR). PCR-amplified DNA fragments were gel-purified and then subjected to DNA sequencing using both forward and reverse primers with the ABI BigDye Terminator Cycle Sequencing Kit v3.1 on an ABI PRISM 3730XL Genetic Analyzer (Applied Biosystems, Inc, Foster City, California, USA). Information about the primer sequences is given in Table 1. The new sequence data has been deposited in GenBank under the accession number KC176705.

**Table 1:** Sequences and positions of the primers used for mutation analysis of *SLC20A2*.

*Amplicon*	*Forward (5′-3′)*	*Reverse (5′-3′)*	*Start*	*End*
SLC20A2-1	CATGCCAAAGTTAGATCCCA	AGAAAATAAATGGTTGCCTGA	42329414	42330282
SLC20A2-2	CGCTTTGTAAAGAAACAATTCACA	GCTCACGCCTATAATCCTG	42322944	42323568
SLC20A2-3	GTCAGCTCTGCCAAGTCA	ACAATTATTCCTCTAACCCCTC	42320428	42320778
SLC20A2-4	CAACAGTGGGCTCTTTGACA	TTACTATCAGCCAACAACTCC	42317264	42317737
SLC20A2-5	TTTAAGCACATATTCGCCAGA	CTTCCAGTTACTCATGGCAAC	42301935	42302488
SLC20A2-6	CCTGGCCTCAACTTCATTTTCTC	CCCCAGTGCCTCCGGTTAG	42296723	42297329
SLC20A2-7	GGCATGGTGTCGCGCTTGTAG	CCGGCGACCTCCTAGCTTGT	42294138	42295291
SLC20A2-8	CCGCGGCTGTAGTCTCAATTA	GGGGCCTGTTTAAGTCTGTGC	42287237	42288029
SLC20A2-9	GCGGCCTCTTGTTCTGTTAAAAT	CCCGGAGACCTGGAGAACCT	42286139	42286509
SLC20A2-10	GCTGAAGAGAAGAATCCCCAAAC	GGTGAACAGTGTGGGATGGAG	42275160	42275667

Genomic position of PCR primers corresponding to the Feb 2009 human genome reference sequence GRCh37.

### Non-denaturing polyacrylamide gel electrophoresis (PAGE)

To confirm the presence of the c.510delA mutation, the PCR amplicons generated with primers delF (5′- GTTTATATCTCCACTGTTGTCTGG -3′) and delR (5′- ACAATTATTCCTCTAACCCCTCTC -3′) were denatured at 95°C for 5 min and annealed by cooling to 25°C (5°C/min) in the thermal cycler. The resultant heteroduplexes and homoduplexes were mixed with 6× gel loading buffer, subjected to electrophoresis in an 8% non-denaturing polyacrylamide gel (30∶0.8 Acrylamide∶Bis), which had been pre-run at 200 V for 30 min. Then, electrophoresis of the samples was performed at 400 V for 4 h. TBE buffer (1×) was pumped to cycle between the upper and lower electrophoresis buffer tanks. DNAs were silver-stained and analyzed visually [9]. All family members and 167 unrelated normal controls were included in the non-denaturing PAGE analysis.

### Analysis of mRNA expression

Total RNA was isolated using the Trizol reagent from the peripheral blood of the family members according to the manufacturer's instructions (Invitrogen, Carlsbad, CA, U.S.A.). Reverse transcription (RT) was performed using a PrimeScript® RT Reagent Kit with gDNA Eraser (Takara, Japan) in a 20 µl reaction mixture containing 1 µg of total RNA from each individual sample. To assess the expression of the mutant *SLC20A2* alleles, RT-PCR was carried out with primers RT-F (5′-AGGTGCCAAGGCTAATGATGAC-3′) and RT-R (5′-GGACAGTGCTCTTCCGTATGC-3′) using RNA from the family members and normal controls. The cDNA fragments produced were then sequenced using the same primers. Quantitative real-time RT-PCR (qRT-PCR) was used to quantify the relative mRNA expression of *SLC20A2* in affected individuals and normal controls. The qRT-PCR primers are given in Table 1. The amplicon of *SLC20A2* that was generated was 124 bp in length, and was normalized to a 114 bp glyceraldehyde-3-phosphate dehydrogenase (*GAPDH*) fragment. The relative expression level was determined on the basis of the comparative ΔΔC_T_ method using the reverse transcription products from peripheral blood samples from a normal individual as calibrators [10]. qRT-PCR was carried out in a total volume of 25 µl, with each tube containing 12.5 µl of SYBR Premix Ex Taq (Takara), 4 µl of reverse transcription product (40 ng) and 2 µl of primers (400 nM each). Three replicates were conducted per sample. Reactions were run in a Rotor-Gene 3000 real-time rotary analyzer (Corbett Life Science) at 95°C for 3 min and then 45 cycles at 95°C for 5 s and 60°C for 20 s. The qRT-PCR experiments were repeated three or four times.

## Results

### Clinical findings in family PJ-IBGC

In this study, we evaluated the available members of the small Chinese IBGC lineage using CT and serum examination, except II:3. The affected individuals showed symmetrical bilateral basal ganglia calcification (Fig. 1B). In this lineage, only one 54-year-old man showed minimal symptoms, specifically changes in mood and declining memory, while everyone else showed no sign of pyramidal or extrapyramidal symptoms. All of the serum examinations were normal for all available individuals in this lineage. No abnormal lactate peak was observed at the basal ganglia (calcified area) or frontal cortex (uncalcified area) in the 4-year-old FIBGC patient (Fig. 1C).

### Identification of a novel *SLC20A2* mutation

**Figure 2:**
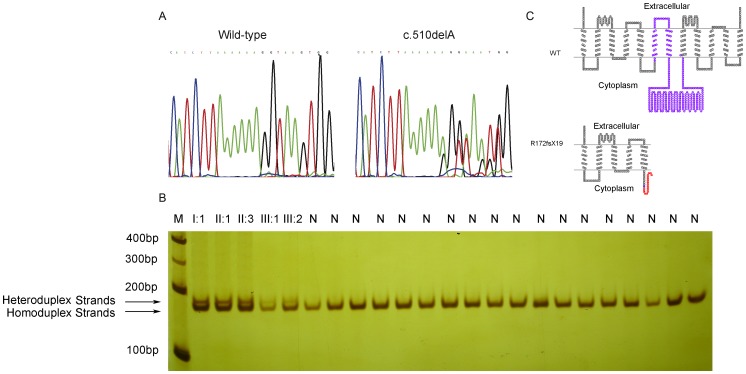
Identification of the c.510delA *SLC20A2* mutation. (A) Sequencing chromatogram showing the heterozygous c.510delA mutation in *SLC20A2* (right) and the wild type sequence (left). (B) Heteroduplex mobility assay of the 171 bp PCR product derived from all the affected individuals and some unrelated normal individuals. After denaturation (95°C), re-annealed reactions were run under non-denaturing conditions. M, 100 bp DNA marker; N, normal individuals. The bands were visualized by silver-staining. (C) Schematic diagram of the wild-type and mutant SLC20A2 proteins. Purple regions represent the L183–V483 fragment of SLC20A2, which are important for the Pi transportation activity. The blue circle indicates the mutated amino acid residue. Amino-acid residues of the novel C-terminal peptides in the p.R172fsX19 mutant are given with the 19 new residues in red. The structure model was drawn using TOPO2 software (http://www.sacs.ucsf.edu/TOPO2/).

Upon screening for *SLC20A2* mutations in the PJ-IBGC family, we identified a heterozygous single base-pair deletion, c.510delA (p.R172fsX19), in *SLC20A2* exon 4 (Fig. 2A). We confirmed the mutation in all affected individuals of the family through DNA sequencing, but did not detect the mutation in any unaffected family members. We then carried out the heteroduplex assay in all family members and 167 unrelated normal individuals. By denaturing and reannealing the PCR products, two types of bands were observed after PAGE. The band at the bottom occurred due to a perfect match of the nucleotide sequences (homoduplex). The upper bands were specific heteroduplex bands, which had a single-nucleotide deletion gap in one relative to a second sequence, and showed reduced mobility in PAGE. Amplicons of all of the affected individuals showed two fragments on non-denaturing PAGE, but only a single homoduplex band was observed in amplicons of all the unaffected and normal controls (Fig. 2B). This indicated that the patients were heterozygous for the specific mutation detected. With this heteroduplex assay, we confirmed that the deletion mutation co-segregated with basal ganglia calcification in all affected family members, but it was not detected in unaffected individuals or in the 167 unrelated controls. The c.510delA mutation, also called p.R172fsX19 at the protein level, results in a frameshift, producing a truncated SLC20A2 protein with a PTC (premature termination codon) (Fig. 2C). Bøttger et al. have shown that the fragment of L183-V483 of human SLC20A2 is critical for Pi transport function [11]. Thus the c.510delA frameshift mutation is likely to lead to a complete loss-of-function of the SLC20A2 protein.

### Detection of allele imbalance from the wild-type and mutant *SLC20A2*

**Figure 3:**
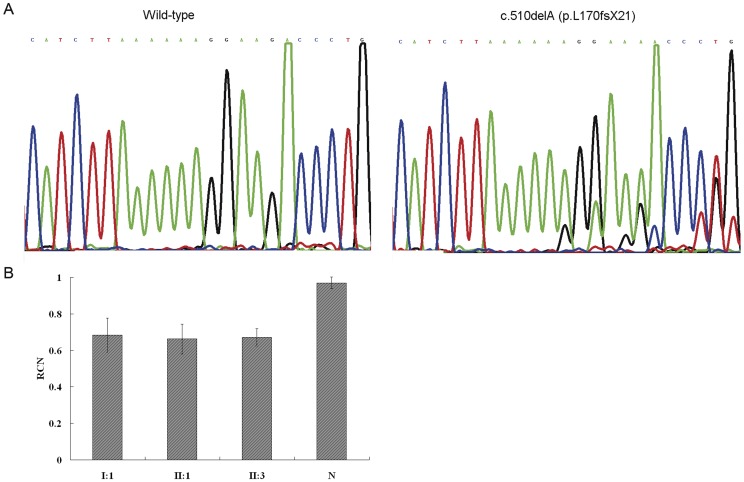
mRNA expression analysis of the *SLC20A2* mutation. (A) Sequencing showing an imbalance of the c.510delA (p.R172fsX19) mutant and wild-type alleles in cDNA templates. (B) Relative quantity (mean ± SD) of *SLC20A2* transcripts derived from real-time quantitative polymerase chain reactions in 3 affected individuals and a normal individual.

To assess *SLC20A2* expression at the mRNA level, RT-PCR fragments spanning the heterozygous mutation were sequenced simultaneously with PCR products derived from genomic DNA of the same affected individuals. In RT-PCR assays, cDNA fragments of the expected size were observed both in normal and affected individuals. No additional bands were detected in the affected individuals, excluding the possibility of exon skipping due to the c.510delA mutation within exon 4 (data not shown). In sequencing, a marked allele imbalance was observed in cDNA from all affected individuals, suggesting lower mRNA expression of the mutant *SLC20A2* allele (Fig. 3A). This imbalance was not detectable in unaffected family members or in unrelated normal controls, which suggested the presence of a difference in copy number between affected and unaffected family members.

### Relative quantity of *SLC20A2* transcripts

To find out whether *SLC20A2* is associated with copy number mutations in the PJ-IBGC family, qRT-PCR was used to determine the relative copy number (RCN) of the *SLC20A2* based on the ΔΔC_T_ method. We examined the 3 affected individuals and an unrelated normal control, and the RCNs were 0.66 (mean ± SD 0.66±0.081), 0.67 (mean ± SD 0.67±0.047), 0.68 (mean ± SD 0.68±0.092) and 0.97 (mean ± SD 0.97±0.032) (Fig. 3B). These data indicate that the *SLC20A2* mRNA expression in peripheral blood samples from normal individuals were constant, but that there was an approximately 30% reduction in cells from affected individuals.

## Discussion

Familial IBGC, also known as Fahr disease, is a rare disorder. Its major clinical features include symmetrical bilateral calcification of the basal ganglia in the absence of any abnormal serum levels of calcium or phosphorus [1]. The patients may show different symptoms, including cognitive impairment, schizophrenia, parkinsonism, and cerebellar ataxia, ranging in severity from undetectable to severe [1], [2].

Dai et al. mapped an IBGC locus to chromosome 8p21.1-q11.23 in a study performed on a large Chinese family [6]. Recently, Dai's research group sequenced candidate genes in this locus and identified several mutations in *SLC20A2* [7]. ^32^Pi transport assays in X*enopus laevis* oocytes showed that the mutations resulted in significant impairment of Pi transport. In the present study, we screened for the *SLC20A2* mutations in the PJ-IBGC family, and identified a novel heterozygous single base-pair deletion, c.510delA (p.R172fsX19), in *SLC20A2* exon 4. The mutation is predicted to result in a frameshift, producing a truncated SLC protein with a premature termination codon, which most likely leads to a complete loss of function in *SLC20A2*. Compared with the work of Wang et al. [7], we performed further confirmation by directly examining the *SLC20A2* mRNA expression in cells from affected individuals and showed a 30% reduction. Our research indicates that insufficient expression of *SLC20A2* is the molecular basis for FIBGC.

Pi is indispensable for all life processes, and Pi homeostasis is precisely controlled at several levels by sodium/phosphate co-transporters. Type-2 sodium/phosphate co-transporters (SLC34) are strongly expressed in the kidneys and intestinal mucosa; they are involved in the absorption of Pi from the intestine and the recycling of Pi from the kidneys [12]. SLC20A2, also known as pit2, is a type III Na-Pi co-transporter. The type III Na-Pi co-transporters are expressed in all investigated tissues and cells of the human body [13]. Both SLC20A1 and SLC20A2 have been used by viruses as receptors for cell invasion [14], [15]. The widespread expression of SLC20A1 and SLC20A2 indicates that they may play a key role in the regulation of Pi homeostasis at the micro-environmental level, and regional differential expression of these two proteins may lead to a different local level of Pi. This may affect Pi-related physical processes, including calcification.

Although a mutation in *SLC20A2* was identified as the genetic basis for the IBGC, several questions still need to be answered: 1. If all of the cells within the body have the same mutation, why does the calcification only occur within the brain? 2. Why does the calcification usually begin in the globus pallidus? 3. Is the location of the calcification consistent with expression of SLC20A2? The expression of SLC20A2 within mouse and human brains was investigated (Allen Brain Atlas, http://human.brain-map.org). The results show that the expression of SLC20A2 was high in regions that usually show calcification in IBGC patients, such as the basal ganglia, cerebral cortex, and cerebellar cortex. However, in some regions, such as the substantia nigra (SN), SLC20A2 was also expressed at high levels, but IBGC patients never show a calcification in this area. For this reason, other factors may also be involved in the calcification process. Villa-Bellosta et al. showed that SLC20A2 function could be regulated by acidosis [16]. We performed MRS to determine the local lactate level. Lactate is a marker of metabolic activity, and abnormal accumulation of lactate may indicate regional acidosis. The 4-year-old girl with minimal calcification was evaluated with MRS, and we did not find any increase in lactate level in the calcified areas or any difference in lactate level between the calcified area and the unaffected area. In addition, other studies have shown no significant regional variations in pH value within the human brain [17]. As a result, acidosis does not seem to be involved in the regulation of Pi homeostasis in the brain.

Pathological analysis of IBGC individuals showed that the calcification of IBGC involved the media and intima of cerebral vessels, including small arteries, small veins and capillaries [18]. This is consistent with the minimal and late onset of the symptoms because no neuronal damage occurs during the early stages of IBGC. Studies on the pathogenesis of vascular calcification induced by phosphate showed that SLC20A1 has a determinant role in the mineralization of vascular cells *in vitro* [19], [20]. For this reason, we propose that both SLC20A1 and SLC20A2 might be involved in the pathogenesis of FIBGC. It has been previously demonstrated that both SLC20A1 and SLC20A2 are expressed in the brain but in different patterns [21]. The basal ganglia area expresses high levels of SLC20A2 but low levels of SLC20A1, which may make this area vulnerable to *SLC20A2* mutation. Researchers have also found that SLC20A2 expression cannot be regulated in the brain, which makes SLC20A2 an ideal target gene for IBGC [21]. Although the SN has an expression pattern similar to that shown by SLC20A1 and SLC20A2 in the basal ganglia, SLC20A1 expression can be up-regulated in this area [21]. This may compensate for the mutation of *SLC20A2* and maintain the local Pi homeostasis. We speculate that, although SLC20A2 is widely expressed outside brain, the co-expressed SLC20A1 may compensate for mutations in *SLC20A2* and render the cells outside of the brain resistant to *SLC20A2* mutations. This may be an explanation for why no calcification takes place outside the brain in IBGC patients. The unique expression of both SLC20A1 and SLC20A2 may explain the specific calcification pattern observed in IBGC.

In conclusion, we identified a novel *SLC20A2* mutation, which causes a significant decrease in *SLC20A2* mRNA expression. Our results indicate that the insufficient *SLC20A2* expression caused by the *SLC20A2* mutation is the molecular basis for the pathogenesis of IBGC and that insufficient SLC20A2 expression may lead to abnormal local Pi homeostasis and then to calcification of the basal ganglia. To explain the unique calcification pattern of IBGC patients, we here propose a hypothesis that the regional expression pattern of SLC20A1and SLC20A2 may be the molecular basis for IBGC. Our hypothesis has partial support from our results and the literature reviews. However, this hypothesis requires further careful investigation.
